# Cultural Interpretations of Patients and Employees in an Organization Certified Through the Baby-Friendly Hospital Initiative: A Focused Ethnographic Study

**DOI:** 10.1177/08903344251337375

**Published:** 2025-05-27

**Authors:** Keri Durocher, Kimberley T. Jackson, Richard Booth, Panagiota Tryphonopoulos

**Affiliations:** 1 Arthur Labatt Family School of Nursing, Faculty of Health Science, Western University, London, ON, Canada

**Keywords:** Baby-Friendly Hospital Initiative, breastfeeding, breastfeeding experience, breastfeeding knowledge, breastfeeding practices, breastfeeding support, COVID-19, cultural norms, ethnography, health services research, postpartum care

## Abstract

**Background:**

When organizations are certified through the Baby-Friendly Hospital Initiative, health care providers implement various policies that are intended to support long-term and exclusive breastfeeding. Despite the availability of evidence to support these policies, research findings are inconsistent in whether these goals are met. Exploring cultural interpretations through the lens of individuals within these organizations may reveal new evidence of breastfeeding experiences and needed support.

**Research Aim:**

To explore organizational cultural aspects of a Baby-Friendly certified organization from the perspective of patients and employees.

**Method:**

Researchers implemented a focused ethnography design in one certified organization in Ontario, Canada. One-to-one, semi-structured interviews were performed with two participant groups, including 10 patients and eight employees within intrapartum and postpartum care areas between 2023–2024. An inductive data analysis approach followed Roper and Shapira’s framework, including (1) coding for descriptive labels, (2) sorting for patterns, (3) identification of outliers, (4) generalizing with constructs and theories, and (5) memoing.

**Results:**

Five core themes emerged from the data, including (1) knowledge is power, (2) community of support, (3) contextual considerations, (4) environment for breastfeeding, and (5) patient factors. Through narrative descriptions, these interrelated themes exhibit how patients and employees have experienced or provided care that is consistent with breastfeeding-supportive policies as well as additional gaps that may not be addressed through policy research.

**Conclusion:**

The results provide implications for breastfeeding support within an organization certified through the Baby-Friendly Hospital Initiative. Understanding cultural interpretations of breastfeeding can provide information for future education and interprofessional development.

## Background

In 1989, the World Health Organization (WHO) and the United Nations Children’s Fund (UNICEF) collaborated to form the Baby-Friendly Hospital Initiative (BFHI), a global breastfeeding program (WHO, 2024). Upon introducing the program in 1991, hospitals could become designated through the implementation of policies, such as the Ten Steps to Successful Breastfeeding (TSSB), intended to support exclusive and long-term breastfeeding (WHO, 2017). As of 2017, the WHO conducted an international survey to discern how many countries had implemented BFHI-specific policies (WHO, 2017). Out of the 117 countries that responded to the survey, approximately 86% had at least one healthcare organization that had implemented the BFHI. However, despite the BFHI’s global reach, its efficacy in enhancing long-term, exclusive breastfeeding has not been well established (Fair et al., 2021; Howe-Heyman & Lutenbacher, 2016). Several studies have argued that these outcomes may be due to how most research has focused on BFHI policy implementation and staff perceptions (Bell, 2016; Howe-Heyman, 2017; C. M. Huang et al., 2016; Hughes, 2015; Pound et al., 2016) rather than how the culture of BFHI-certified organizations influences the experiences of individuals within each organization, including patients and employees.

### Organizational Culture in BFHI-Certified Settings

The concept of culture has been taken up in various ways within health care disciplines to describe different aspects of human society. Organizational culture is a type of subculture within an institutional setting, which can include a variety of individuals that have a connection to the organization (e.g., patients and employees). This type of culture can have a profound effect on the interpretations of individual experiences of those who are a part of this culture (Cruz & Higginbottom, 2013). If researchers can elicit interpretations of individuals within specific organizational cultures, then the complexities of cultural phenomena can be better understood (Fetterman, 2010; Roper & Shapira, 2000).

There is a lack of research on cultural perceptions of how BFHI practices shape individual experiences within these organizations. To date, one study from the United Kingdom has explored the cultural perceptions of both staff and patients within a BFHI-certified institution using critical ethnography methods (Byrom et al., 2021). The researchers present a broad sociological understanding of the BFHI; however, their findings may not apply to other global contexts, as the study was conducted at a single site within a unique healthcare system. A second ethnographic study, which took place in a BFHI-certified organization in the Colombian Caribbean, explored mothers’ understanding of breastfeeding benefits but did not include any information on cultural interpretations of their in-hospital experiences that could affect breastfeeding (Echevarría et al., 2020). Lastly, findings from qualitative studies have explored individual attitudes about breastfeeding within BFHI organizations; however, specific connections to BFHI cultural interpretations were not included (Howard et al., 2022). Without a deeper understanding of these cultural interpretations and operationalization of BFHI-specific policies and values in organizations, there may be a lack of patient-centered and therapeutic care for breastfeeding support. Therefore, the purpose of this study was to explore organizational cultural aspects from the perspective of patients and employees.

## Methods

### Research Design

We used a focused ethnographic design to examine the cultural interpretations of individuals within a BFHI-certified organization. Ethnography is a method that promotes a comprehensive view of a culture (Cruz & Higginbottom, 2013; Polit & Beck, 2016; Rashid et al., 2019) where researchers spend time with individuals who are a part of the culture of interest within their natural settings and learn from their interactions (Higginbottom et al., 2013; Roper & Shapira, 2000). This design is a sub-methodology to conventional ethnography when narrowed issues or sub-cultures are within a researcher’s interest (Cruz & Higginbottom, 2013; Fetterman, 2010) and is sometimes referred to as microethnography (Polit & Beck, 2016) or mini-ethnography (Leininger, 1985). A focused ethnography design is useful in health care contexts, as people within the subculture have specific knowledge about the topic of interest, and researchers can elicit this information for priority health applications (Cruz & Higginbottom, 2013; Higginbottom et al., 2013). Additionally, researchers spend more time on recording and communicative activities, rather than conducting long-term field visits (Knoblauch, 2005). This study received ethical approval from the Research Ethics Board at Western University (Project ID# 121479), and from the participating hospital (ID#1186) on March 23, 2023, and August 1, 2023, respectively.

**Key Messages**

- The Baby-Friendly Hospital Initiative is a global program implemented to enhance breastfeeding outcomes, including exclusivity and longevity. However, previous research in these areas has not been conclusive as to whether the intended goals are met. Understanding experiences of breastfeeding from a patient and employee perspective can provide further information on potential care gaps.
- In addition to Baby-Friendly policies, organizations should consider other strategies to enhance patients’ knowledge of breastfeeding, and ensure they are connected to resources for breastfeeding support after discharge.
- This study provides additional considerations in addition to Baby-Friendly policies that may be useful when planning breastfeeding education and development activities.

### Setting and Relevant Context

This study was set in a BFHI-certified, urban hospital organization in the Greater Toronto Area region of Ontario, Canada. This organization provides comprehensive health care to women and children, serving a population of almost 1 million people. Ontario is the most populous Canadian province, with a population of approximately 15.5 million (Government of Ontario, 2023). As of 2018, eight out of 94 hospitals providing maternity services in Ontario were BFHI-certified (Baby-Friendly Initiative Ontario, 2019). As of 2022, Ontario’s breastfeeding initiation rate was 92.1%, compared to the national rate of 91.1% (Public Health Agency of Canada, 2022). Additionally, 64.6% of people continue breastfeeding beyond 6 months of life, while 36.3% breastfeed exclusively. Contextual factors may increase Canada’s breastfeeding rates compared to other countries. For example, individuals who contribute to a national employment insurance program can receive 12–18 months of paid maternity/parental leave (Government of Canada, 2023).

### Sample

The two target populations for this study included (1) patients who received intrapartum and postpartum health care within the study setting and (2) employees within the study setting. Inclusion criteria for both groups included (1) able to read and speak English; (2) aged 18 or older; and (3) access to a digital device (smartphone, computer, etc.) for a virtual interview. Specific inclusion criteria for the patient group included: (1) received intrapartum/postpartum health care at the BFHI-certified organization within the past 2 years; and (2) initiated breastfeeding. Inclusion criteria for the employee group included (1) current employment within the BFHI-certified organization and (2) working directly with patients (e.g., providing patient care) or being indirectly involved with the BFHI, such as through policy development.

Sampling techniques included purposive and convenience methods, which have been implemented in other studies that included a focused ethnographic design (Cruz & Higginbottom, 2013). We aimed to recruit approximately 20 participants; this estimation was determined based on sample sizes from recent focused ethnographic studies within health contexts (Calloway & Copeland, 2021; Kalogirou et al., 2021; Yantzi et al., 2023). The final sample size was 18, including 10 patients and eight employees. Patient recruitment strategies included using a digital recruitment poster on the social media platforms Meta and X. Recruitment of employees included sending an organizational email that included information about the study and the digital poster, as well as placing hardcopy posters in pertinent units, including labor and delivery, postpartum, and neonatal intensive care (NICU). The recruitment posters listed the study’s purpose, inclusion criteria and contact information for potential enrollment. Participants understood the study’s purpose as recognizing experiences within a BFHI-certified organization with breastfeeding (patients) or providing direct (point of care) or indirect (e.g., policy implementation) breastfeeding support (employees). Each participant received a $50 CAD ($36 USD) gift card as an honorarium via email after completing the interview. The sample size was deemed adequate when data saturation was reached, as no newly emerging information was gained from the interviews (Guest & Namey, 2020).

### Data Collection

Data collection methods included individual interviews that occurred between April 2023 and January 2024. Potential participants contacted the research team by email, where the team members confirmed that they met the eligibility criteria. Participants were then sent a link, which included a demographic survey (see the online Supplemental Material File 1), and the informed consent form. After reviewing the study’s purpose, procedures, potential risks/benefits of enrollment, and maintenance of confidentiality procedures, potential participants signed the letter of informed consent to enroll in the study. The research team signed the form and sent the participant the completed copy. Interviews lasted 30–60 minutes and were scheduled on a flexible basis. The primary author, a Registered Nurse (RN) and PhD in nursing student with 8 years of clinical experience in postpartum/pediatric care settings, conducted all interviews via Zoom videoconferencing. Participant confidentiality was maintained through safeguarding methods, including using a waiting room and a password to access each videoconferencing session.

Individual interview guides were developed for both participant groups, which included topic guides and semi-structured questions. Topics, sample questions and prompts were developed by the primary author and the Principal Investigator (PI), a PhD-prepared RN who has done widespread research in breastfeeding and perinatal care areas. Both descriptive and structural questions were asked throughout the interviews (Merkle Sorrell & Redmond, 1995). To view the full interview guides, please see the online Supplemental Material. Due to the primary author’s familiarity with BFHI policy implementation and her potential influence on participants, discursive reflexivity was implemented during data collection procedures (Cooper & Burnett, 2006). This helped the research team reflect on their assumptions and understand how participants’ communication related to their experiences (Carbaugh et al., 2011). Additionally, participant confidentiality was strictly maintained, as all data were anonymized directly following each interview by assigning each participant a unique identifier. Therefore, the only records that could identify each participant were the letters of informed consent and a master list. These forms were kept in a secure, password-protected server through Western University’s OneDrive.

### Data Analysis

Directly following data collection, interviews were anonymized and transcribed by a professional transcription service. An inductive approach to data analysis was employed, utilizing Roper and Shapira’s (2000) method for analyzing ethnographic data, which included (1) coding for descriptive labels, (2) sorting for patterns, (3) identification of outliers and negative cases, (4) generalizing with constructs and theories, and (5) memoing including reflexive remarks. The primary author and PI performed all analysis procedures. To enhance the trustworthiness of this study, the research team employed data triangulation to review the data from different viewpoints and enhance the reflexive process (Lincoln & Guba, 1985; Rashid et al., 2019). The process of coding was iterative, as the two researchers had meetings to discuss coded labels, constructs, and outliers. Similar constructs were then grouped into categories, which became themes based on similarities. The finalized themes were connected to the research question using exemplar quotes from the participants. Please see Table 1 for the data analysis structure.

**Table 1.** Data Analysis Structure.

Theme	Theme Definition	Categories	Category Definitions	Participants Contributing (*N* = 18)
Knowledge is Power	How knowledge can enhance breastfeeding experiences within a BFHI-certified organization.	1. Resource availability 2. Hands-on education 3. Preparatory knowledge 4. Process of learning 5. Staff expertise	1. Accessibility of materials for breastfeeding education. 2. One-to-one education and assistance from health care providers. 3. Patients’ breastfeeding knowledge prior to giving birth. 4. Education as an ongoing process and the recognition of knowledge gaps. 5. Breastfeeding knowledge and skills of employees.	17
Community of Support	How the support of various members of the community can contribute to breastfeeding experiences.	1. Discharge coordination 2. Organizational championship 3. Interdisciplinary teamwork 4. Staff attitudes 5. Compassionate care 6. Family as collaborators	1. Connecting patients to resources after discharge from the hospital. 2. Promoting awareness of the BFHI throughout the organization. 3. Collaboration by multiple health care providers for promotion of breastfeeding. 4. Attitudes about breastfeeding by employees. 5. Displays of empathy by health care providers while providing care. 6. Involving family members and care partners in supportive breastfeeding care, as desired by the patient.	17
Contextual Considerations	How varying circumstances can affect breastfeeding experiences, despite BFHI implementation.	1. Health implications 2. Pandemic-related barriers 3. Workload	1. Patient or infant health complications that can affect breastfeeding. 2. Implications of the COVID-19 pandemic that negatively affect breastfeeding experiences. 3. How breastfeeding support can be affected by increased staff workload.	13
Environment for Breastfeeding	The environmental considerations that contribute to breastfeeding experiences.	1. Physical environment 2. Unit coordination 3. Normalization of practices	1. Factors within the physical environment that can promote breastfeeding. 2. Collaboration between patient units to promote breastfeeding. 3. Engraining breastfeeding support into regular care practices.	10
Patient Factors	Patient circumstances that contribute to their breastfeeding experiences.	1. Emotional responses 2. Determination 3. Supporting choice	1. The variety of emotions patients can experience related to breastfeeding. 2. Positive feelings that are motivational for breastfeeding. 3. Employees being accepting and supportive of patient’s feeding choices.	14

## Results

### Characteristics of the Sample

Eight employees and 10 patients participated in this study. All the participants identified as female. Most of the employee participants were RNs and had been employed by the BFHI-certified organization for 11–20 years. Most of the patient participants were between 30–40 years old, held a bachelor’s degree, and had a family income of 100,000–200,000 CAD (72,000–145,000 USD). Full details of the characteristics of the sample are listed in Tables 2 (Employees) and 3 (Patients). All the patient participants reported having breastfeeding support from their partners and their communities. Most of the participants exclusively breastfed (no introduction of a human milk substitute) for the total duration of breastfeeding. Full details of the patients’ breastfeeding characteristics are listed in Table 4.

**Table 2.** Characteristics of Participants – Employees (*N* = 8).

Characteristic	*n* (%)
Age	
≤ 29	2 (25)
30–39	1 (12.5)
40–49	4 (50)
≥ 50	1 (12.5)
Gender Identity	
Female	8 (100)
After-Tax Yearly Family Income (CAD)	
50,000–99 999	2 (25)
100,000–149 999	2 (25)
150,000–200 000	3 (37.5)
Prefer not to answer	1 (12.5)
Professional Role or Designation	
Registered Nurse (Staff)	5 (62.5)
Clinical Nurse Educator	2 (25)
Registered Respiratory Therapist	1 (12.5)
Years Employed in BFHI Organization	
≤ 10	2 (25)
11–20	5 (62.5)
21–30	1 (12.5)

*Note.* CAD = Canadian dollars, BFHI = Breastfeeding Friendly Hospital Initiative. Conversion - $1 USD = $1.37 CAD.

**Table 3.** Characteristics of Participants – Patients (*N* = 10).

Characteristic	*n* (%)
Age	
≤ 29	1 (10)
30–35	5 (50)
36–40	3 (30)
≥ 41	1 (10)
Gender Identity	
Female	10 (100)
Race	
White/Caucasian	7 (70)
Asian	1 (10)
South Asian	1 (10)
White/Metis	1 (10)
Ethnicity	
Canadian	3 (30)
Western European	3 (30)
Filipino	1 (10)
Pakistani	1 (10)
Prefer not to answer	2 (20)
After-Tax Yearly Family Income (CAD)	
50,000–99 999	1 (10)
100,000–149 999	4 (40)
150,000–200 000	4 (40)
> 200 000	1 (10)
Underlying Health or Medical Conditions	
No	8 (80)
Yes	2 (20)
Highest Level of Completed Education	
Bachelor’s degree	7 (70)
Master’s or other advanced degree	3 (30)

*Note.* CAD = Canadian dollars. Conversion - $1 USD = $1.37 CAD.

**Table 4.** Breastfeeding Characteristics – Patients (*N* = 10).

Characteristic	*n* (%)
Previous breastfeeding experience	
Yes	5 (50)
No	5 (50)
Partner support with breastfeeding	
Yes	10 (100)
No	0 (0)
Community (family, friends, etc.) support with breastfeeding	
Yes	10 (100)
No	0 (0)
Total duration of any breastfeeding (with or without supplementation)	
3–6 months	3 (30)
7–12 months	2 (20)
13–24 months	1 (10)
> 24 months	1 (10)
Ongoing at time of interview	3 (3)
Breastfeeding exclusivity (no introduction of human milk substitute)	
Yes	9 (90)
No	1 (1)

### Themes

**Figure 1.**
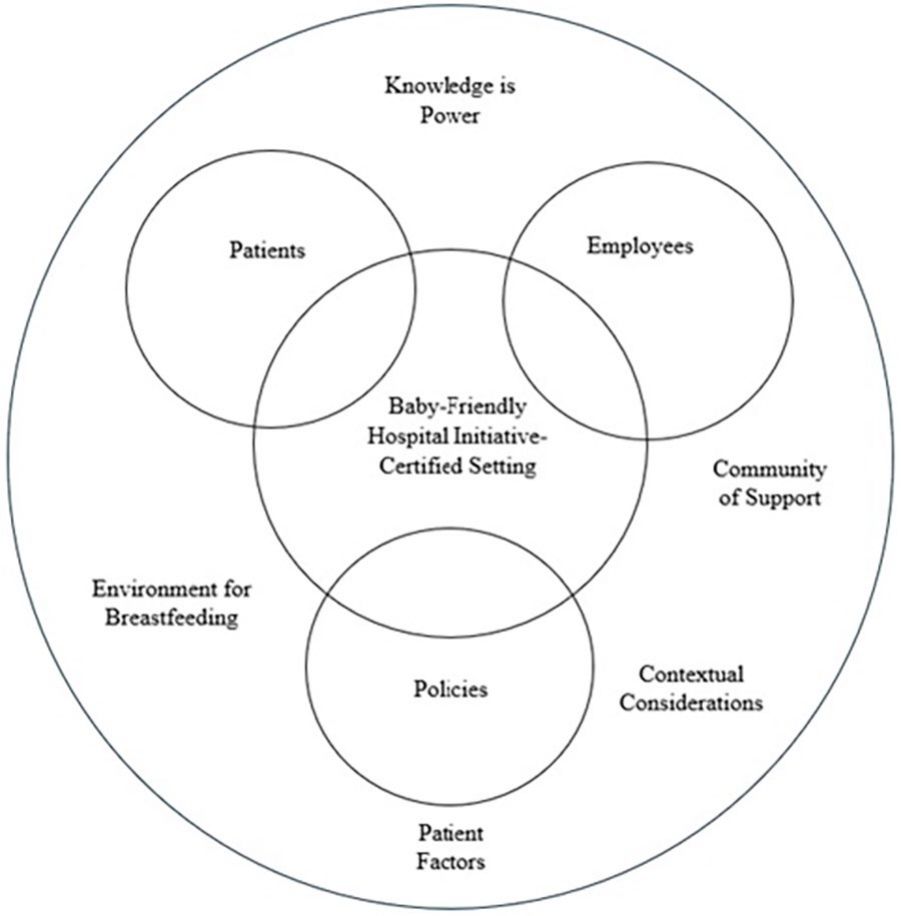
Organizational Culture Within a Baby-Friendly Hospital Initiative-Certified Setting.

Results of the analysis included five key themes: (1) knowledge is power, (2) community of support, (3) contextual considerations, (4) environment for breastfeeding, and (5) patient factors. The organizational culture of a Baby-Friendly Hospital Initiative-certified setting is represented in Figure 1.

#### Knowledge is power

Participants described how the variety of resources available to support breastfeeding was integral to an organizational culture of breastfeeding support. Resources for patients ranged from information about “skin to skin” (Patient Participant 104) to how colostrum is like “liquid gold.” (Patient Participant 105). The importance of this information was described as “supporting patient learning and education” (Employee Participant 112) and to inform patients about the benefits of breastfeeding.

We have a little chart where we have all the benefits of breastfeeding. Like everything that’s in the breast milk and then everything that’s in the formula and then you’ll see visually the ingredients in the formula are like here and breastfeeding has a lot more nutrients. So, it helps them (patients) visualize as well and they get more motivated to store their milk for baby. (Employee Participant 113)

The availability of human resources and hands-on support was also described as important by many participants within the organizational culture. This support ranged from a formal “breastfeeding class” (Patient Participants 109, 110) to one-on-one “feedback” (Patient Participant 110) from nurses and Internationally Board-Certified Lactation Consultants (IBCLCs). The comprehensiveness of this education within the NICU was described by Employee Participant 118:

Educating is huge too. These moms have to be there as much as possible, talking to them about the frequency of feeding, the frequency of their pumping, the frequency of how we give oral immune therapy through the colostrum. Educating them on the composition of colostrum, why it’s so good. Foremilk, hindmilk, all of that.

Conversely, participants noted how a lack of human resources can be detrimental to organizational culture, specifically a lack of IBCLCs. The need for “more” lactation support (Patient Participant 103, Employee Participant 115) due to IBCLC’s “support of [breastfeeding] initiatives” (Employee Participant 117) was described by a variety of participants. However, the expertise of other staff members was significant in enhancing the experiences of both patients and employees and could help bridge this gap. From two employee’s perspectives, “watching” (Employee Participant 117) or being “paired up” (Employee Participant 113) with experienced staff members developed their own competence in providing breastfeeding support. Patient Participant 109 described her experience of receiving breastfeeding support from a nurse:

I really appreciated that the nurse, that even though she wasn’t an educator, she was competent enough in breastfeeding. That she was aware that there was a struggle and what would’ve been the best sort of, I don’t know, treatment plan or sort of what would have helped facilitate breastfeeding.

Having adequate preparatory knowledge prior to birth was described by many participants as integral to positive breastfeeding experiences. Patient participants remarked how they did “research” (Patient Participant 106) and sought information through “reading” (Patient Participant 105) and attending “prenatal workshops” (Patient Participant 101). Some of this knowledge was experiential from breastfeeding previous children

They put them on me, and I didn’t really know much with my first. And I was kind of indifferent. I didn’t really know about breastfeeding that much. I was just like, “I’ll try it.” And they taught me a ton. And then with my second, I felt super comfortable, and I knew kind of what to do right away. (Patient Participant 107)

Despite some patient participants having preparatory knowledge, the process of learning was ongoing during the initiation of breastfeeding. One participant noted how she “did not do enough research” (Patient Participant 107). Employees also agreed that some patients are “more educated [in breastfeeding] than others” (Employee Participant 117) and that “a lot of education needs to be done” (Employee Participant 113).

#### Community of support

Fostering a community of support within a BFHI-certified organization was seen as imperative to positive organizational culture in a variety of ways. The importance of discharge coordination for breastfeeding support was discussed by multiple participants, including information on resources such as La Leche League (Patient Participant 102) and Public Health (Patient Participant 109). Patient Participant 108 described the importance of these resources:

I think there’s so much information out there that it sometimes can be overwhelming, especially when you come home with a newborn that you’re expected to take care of yourself. So, it’s good to just get a handout or something that tells you, this is exactly what you should be following or what you can do if this is what you want, this is your goal.

Including family members and care partners as collaborators was also seen as a cultural norm. Employee Participant 112 remarked how it should be the “standard to include them [family members]” especially when they will be involved in supporting breastfeeding upon discharge: “If their support people are grandparents or sisters or aunts or things like that, making sure they’re involved in that conversation and that education, or discharge teaching, or whatever stage they are there for.”

Within a community of support, participants also emphasized the importance of teamwork and compassionate care. One participant remarked how she felt that care was delivered on a “personal level” (Patient Participant 105), and an employee explained how one can, “feel good when [breastfeeding] works” (Employee Participant 115). Employee Participant 116 explained the process of a team approach to supporting breastfeeding in the NICU:

I noticed in rounds, it’s one of those questions that are brought up as a standard, whether or not the child is going to be breastfed, have we gotten consent for donor milk. It’s become standard work for the interprofessional team. So, it’s affected your nurse practitioners and occupational therapists, and physicians, including RNs, of course.

Participants also commented on how the BFHI must be promoted to uphold organizational championship and a supportive breastfeeding culture. Employee Participant 115 remarked, “It’s all over the hospital. Big giant posters. We’re breastfeeding, we’re Baby-Friendly. You can breastfeed anytime, anywhere.” Informing patients of how the organization promotes this initiative was viewed as important by Employee Participant 118:

It’s supporting patients, and the development of children, of babies, properly. As we know, the health benefits of that. And so, it allows the organization to say they’re supportive of something that has been well researched as being the best for newborns, and having that kind of gold standard, and that you really care.

#### Contextual considerations

Understanding the context of specific situations was described by participants as integral to organizational culture. From an employee perspective, workload must be considered as a critical barrier to upholding BFHI-specific policies. As Employee Participant 114 stated, “I feel like it added to our workload, so sometimes it’s hard to do the Baby-Friendly Initiative, and sometimes that takes a backseat.” Implementing BFHI policies was also described as sometimes “arduous” (Employee Participant 116) and can be time-consuming. Employee Participant 118 explained,

If I have three babies and all three of them are term, and they all have latching difficulties, it’s very hard for me to divide myself and give my full BFI attention to each individual one, every 3 hours. That’s my biggest challenge.

As this study was conducted directly following the COVID-19 pandemic, context was provided by multiple participants on how it affected their breastfeeding experiences or ability to provide care within an organizational culture that typically promotes breastfeeding. Employee Participant 111 described how some supportive practices were “backburned.” Care coordination was also affected, as explained by Employee Participant 112:

You had to put all your PPE on and go in rooms, so you kind of tried to bundle your care. And that meant that there wasn’t as much spontaneous attendance in rooms because it took you so long to get in and out of each room. You had to be a bit more thoughtful about planning and spending your time.

Health implications were also discussed specific to patients and their infants and how this must be considered within the organizational culture. Some notable infant conditions included weight loss (Patient Participants 106, 108), hypoglycemia, prematurity, and other factors causing separation. Employee Participant 113 described difficulties in upholding hypoglycemia protocols within the organization:

I think it can be challenging sometimes, especially with the other protocols. For example, the hypoglycaemia protocol, a lot of the times, even if the sugar is borderline or below 2.5, we have to encourage the formula as per the protocol. And a lot of times the mothers don’t want to, they want to stick to breastfeeding. But at that time, it can be challenging because it’s more important to make sure the baby’s sugars are stable.

Maternal health implications that can interfere with breastfeeding ranged from mild to severe circumstances. For example, Patient Participant 109 explained, “I have an inverted nipple, even the nurse sort of noticed that but she was sort of still trying to support me.” However, Employee Participant 115 described severe cases in which implementing some BFHI principles may not be possible,

And there are some extreme cases that moms end up in ICU straight from delivery, and that baby gets bottle fed with the formula, because that mom is fighting for her life. And we have to remember about those moms, too. (Participant 115)

Participants recognized the importance of considering these health implications within an organizational culture that supports and promotes exclusive breastfeeding.

#### Environment for breastfeeding

An environment that is favorable to breastfeeding was viewed as an important cultural consideration for most of the participants. For example, the normalization of breastfeeding practices emerged as a cultural consideration, which participants described as “second nature” (Employee Participant 111) and “automatic” (Employee Participant 115). Physical environments for promoting breastfeeding were also described as beneficial, for example, through “rooming in” (Patient Participant 105) which is a clinical component of the BFHI. Coordination between units was also described through a process of “transitional care” (Employee Participant 115). Employee Participant 112 thoroughly described this process:

Right from the beginning of when they get admitted we talk about their feeding choices so that we can provide any discussion or information or have that conversation right from when they come. So then when they deliver, we’re promoting and supporting their feeding options then too, right? And then continuing that postpartum where they’re going to get a lot more, because baby’s here now, and then that ongoing education postpartum.

Patient Participant 103 described in more detail how coordination occurs between the postpartum unit and NICU: “They mostly showed me how to express my milk, like, my colostrum. And then, it was in the NICU that I kind of had, you know, people showing me how to feed the baby.” This care coordination was deemed imperative across the organization to promote optimal breastfeeding support.

#### Patient factors

Emotional responses to breastfeeding were described by participants as inherent to their cultural experiences. Two participants stated how breastfeeding processes can make individuals feel “overwhelmed” (Patient Participant 103, Employee Participant 113) despite being within a BFHI-certified organization. Patient Participant 104 described this phenomenon from an internal perspective, “It’s like your own hormonal, it’s your societal expectations, it’s your hormones. It’s what you’ve dreamt of or imagined your entire life going completely wrong, and not following through in any type of way that you anticipated.”

However, determination to breastfeed was an internal factor described by multiple participants. Patient Participant 101 remarked, “This was my second child, and my first breastfeeding journey was not successful. I was determined to breastfeed this baby.” Patient Participant 106 described a similar sentiment:

I delivered twins. So, I did a lot of research on breastfeeding before going into it because I had a lot of people around me telling me that it was pretty unlikely that I’d be able to breastfeed them, and I was fairly determined to be able to do it.

Most employees also described that despite the organizational culture of breastfeeding support, patient choice still had to be respected. As described by Employee Participant 113, “It is the patient – parent’s decision. If they have decided [to use formula] even if it’s not medically indicated.”

## Discussion

Our research question was “What are the cultural interpretations of individuals within a BFHI-certified organization?” This study demonstrated that patients and employees had numerous views about the organizational culture within a BFHI-certified organization. The diversity of participants and the applicability of the data to clinical contexts all provided strength to our outcomes.

Previous research has established how breastfeeding rates vary between people of different ethnicities (Quintero et al., 2023), socioeconomic statuses (Foster et al., 2023), and with different health considerations, such as disabilities (Brown et al., 2023). For the patient participants, a variety of demographic data was collected to report on various influences of breastfeeding experiences. For example, our study participants identified with ethnicities, including Filipino, Pakistani, and Western European. The patient participants’ after-tax yearly family income (CAD) also varied widely, and two patient participants identified as having an underlying medical condition. This diversity in the identity of study participants resulted in outcomes reflecting a variety of perspectives that provided a deeper understanding and engagement with organizational culture. All the patient participants stated that they had support from a partner and their community, both of which are known contributors to positive breastfeeding outcomes (Davidson & Ollerton, 2020; Woods Barr et al., 2021). Half of the patient participants also had previous breastfeeding experience, which can influence future breastfeeding outcomes and perceptions (Y. Huang et al., 2019).

One significant finding from this study was that participants spoke about prenatal education enhancing breastfeeding knowledge which improved other aspects of organizational culture, such as workflow. Results from a systematic review by Wouk et al. (2016) echo the importance of prenatal education, which they found to be associated with enhanced breastfeeding exclusivity, duration and initiation in alignment with BFHI policies. A more recent systematic review by Kehinde et al. (2023) also found that prenatal education was associated with an enhancement of a variety of factors, including maternal breastfeeding self-efficacy. Researchers may focus future studies on experiences of prenatal education and breastfeeding in alignment with BFHI policies.

Most participants also discussed the importance of discharge planning as a way of connecting to community resources for breastfeeding support. This finding was especially relevant in the context of the COVID-19 pandemic, which participants described as a time when the availability of these resources was significantly reduced. Results of a recent cross-sectional study support these findings, as participants who gave birth in the first year of the COVID-19 pandemic had lower satisfaction levels than participants who delivered in 2019 (Oggero & Wardell, 2022). A recent quasi-experimental study has shown the effectiveness of providing community resources to support ongoing breastfeeding through a 6-week breastfeeding support program, which reduced breastfeeding cessation rates and promoted exclusivity (van Dellen et al., 2019). Ongoing research might consider the effectiveness of these programs when specifically delivered in alignment with the BFHI.

## Limitations

This focused ethnography study is one of the first to explore cultural interpretations of patients and employees within a BFHI-certified organization. Despite following a rigorous methodological design, this study is not without limitations. First, this study was conducted in one urban hospital system in Ontario, Canada, and therefore the findings may not be generalizable or applicable to all global settings. Second, convenience/purposive sampling methods were used, which we believe limited our participant pool to those who identified as female. Therefore, experiences of males and individuals who identify as gender-diverse (e.g., transgender, non-binary or two-spirit) were not included in this study. Lastly, although a range of employees were invited to participate, most of the participants were RNs. Therefore, researchers conducting future studies may utilize maximum variation sampling methods to capture the experiences of more individuals employed in BFHI-certified hospitals.

## Conclusions

Exploring the experiences of patients and employees within a BFHI-certified organization provides a greater understanding of organizational culture. Specifically, insights on the importance of preparatory breastfeeding knowledge, a supportive community, contextual elements, and patient factors that affect breastfeeding must be considered in alignment with BFHI policy implementation. To continue strengthening breastfeeding support within organizations, future clinical initiatives and education programs should include these considerations to promote a holistic approach to breastfeeding care.

## Supplemental Material

sj-docx-1-jhl-10.1177_08903344251337375 – Supplemental material for Cultural Interpretations of Patients and Employees in an Organization Certified Through the Baby-Friendly Hospital Initiative: A Focused Ethnographic Study Supplemental material, sj-docx-1-jhl-10.1177_08903344251337375 for Cultural Interpretations of Patients and Employees in an Organization Certified Through the Baby-Friendly Hospital Initiative: A Focused Ethnographic Study by Keri Durocher, Kimberley T. Jackson, Richard Booth and Panagiota Tryphonopoulos in Journal of Human Lactation
