# Supplementary material for: Cultural Interpretations of Patients and Employees in an Organization Certified Through the Baby-Friendly Hospital Initiative: A Focused Ethnographic Study
Source: J Hum Lact. 2025 May 27;41(3):423–33. doi: 10.1177/08903344251337375 (PMC12238666; doi:10.1177/08903344251337375)
Supplement: sj-docx-1-jhl-10.1177_08903344251337375 – Supplemental material for Cultural Interpretations of Patients and Employees in an Organization Certified Through the Baby-Friendly Hospital Initiative: A Focused Ethnographic Study [file sj-docx-1-jhl-10.1177_08903344251337375.docx]

**Interview Guide-Employee Participants**

**Introductory Topics and Sample Question**

- Can you describe your experiences working in a BFHI-certified organization?
- How would you describe your role in implementing the BFHI?

***Example Prompts***

- *Sample prompt question: Do you have any thoughts or opinions to share regarding BFHI implementation?*
- *Sample prompt question: What components of ongoing BFHI implementation do you feel are going well?*
- *Sample prompt question: What components of ongoing BFHI implementation pose challenges or are more difficult to implement?*

**Second Topic and Sample Question**

- Referring to the TSSB, can you describe how any of these elements relate to the delivery of patient care?

***Example Prompts***

- *Sample prompt question: Do you believe that any of the clinical practices impact the breastfeeding experiences of patients?*
- *Sample prompt question: Can you share your experiences related to implementing or developing these clinical practices within your work setting?*
- *Prompt: If the participant only lists 1-2 aspects, the other 8 aspects can be briefly reviewed to see if there is any additional information that the participant wants to add.*

**Third Topic and Sample Questions**

- How does working in a BFHI-certified organization impact your workflow?

***Example Prompts***

- *Sample prompt question: Are there any specific components of the TSSB that influence your work duties?*
- *Sample prompt question: Do any specific policies or procedures related to the BFHI influence your work practices?*
- *Sample prompt question: Does the BFHI impact your daily interactions with your colleagues?*
- *Sample prompt question: How do you feel patients perceive BFHI implementation practices?*

**Fourth Topic and Sample Questions**

- How do you feel the implementation of the BFHI impacts your organization and your colleagues?

***Example Prompts***

- *Sample prompt question: Do you feel there is a collective effort among all employees in delivering the BFHI?*
- *Sample prompt question: Do all employees understand the rationale for BFHI implementation?*
- *Sample prompt question: How does the implementation of BFHI policies differ from previous work practices? (This prompt only relates to participants who were within the organization prior to the implementation of the BFHI).*
- *Sample prompt question: Do you feel that any components of the BFHI can be improved to better suit the needs of your organization?*

**Interview Guide- Patient Participants**

**Introductory Topics and Sample Questions**

- Can you describe your breastfeeding experience while in-hospital?
- What does your breastfeeding experience mean to you?

***Example Prompts***

- *Sample prompt question: How did the health care providers help you breastfeed?*
- *Sample prompt question: How did you feel during the initial stages of breastfeeding?*

**Secondary Topics and Sample Questions**

- Can you expand on how *(xx topic)* was influential in your breastfeeding experience while in-hospital?

***Example Prompts***

- *Sample prompt question: You mentioned that you had to implement a human milk substitute on day two of your hospital stay, can you tell me more about this experience?*
- *Sample prompt question: You remarked how you enjoyed the rooming in process. Can you explain more about this experience?*
- *Prompt: You said you wished that you were able to give your infant a pacifier while in hospital. How did this experience make you feel?*

**Third Topic and Sample Question**

- Referring to the TSSB, can you describe if any of these other elements were relevant to your hospital experience?

***Example Prompts***

- *Sample prompt question: Were you counselled on any potential risks of introducing bottles for feeding?*
- *Sample prompt question: How did you feel about the rooming-in experience with your infant?*
- *Sample prompt question: Were there any difficulties that you experienced with feeding?*

**Final Topic and Sample Questions**

- Now that you have reflected on your hospital experiences with the BFHI, do you think that any parts of the TSSB impacted your breastfeeding practices after discharge?
- Are there any other barriers or facilitators that have impacted your breastfeeding experience, other than the BFHI/TSSB components?

***Sample Prompts***

- *Prompt: The participant can be asked about their breastfeeding experience after discharge. I can then ask if any of these aspects are related to the participant’s in-hospital experience. Specific hospital experiences that the participant mentioned can be reflected on to see if this helps the participant makes connections.*

**Demographic Questionnaire- Employee Participants**

Q1 What is your age?

________________________________________________________________

Q2 What gender do you identify with?

________________________________________________________________

Q3 What is your average, after-tax yearly family income (CAD)?

________________________________________________________________

Q4 What is your professional role or designation?

________________________________________________________________

Q5 How many years have you worked in your current institution?

________________________________________________________________

Q6 How many years have you worked in your profession (current institution or other)?

________________________________________________________________

**Demographic Questionnaire and Breastfeeding History- Patient Participants**

Q1 What is your age? ________________________________________________________________

Q2 What gender do you identify with?

________________________________________________________________

Q3 What race(s) do you identify most closely with?

________________________________________________________________

Q4 What ethnicity (or ethnicities) do you identify most closely with?

________________________________________________________________

Q5 What is your average, after-tax yearly family income (CAD)?

________________________________________________________________

Q6 Do you have an underlying health or medical condition?

- No (1)
- Yes (please list if you feel comfortable doing so) (2) __________________________________________________

Q7 What is your highest level of completed education?

- Some high school (1)
- High school (2)
- College diploma (3)
- Bachelor's degree (4)
- Master's or other advanced degree (5)
- Doctoral degree (6)

Q9 Have you returned to work or do you plan on returning to work?

- Yes (1)
- No (2)

Q10 If you answered "yes" to the previous question, how many months after birth did you return to work, or are you planning on returning to work?

- Less than 3 months (1)
- 3-6 months (2)
- 7-9 months (3)
- 10-12 months (4)
- 13-18 months (5)
- Greater than 18 months (6)

Q11 Prior to your most recent birth experience, did you have experience breastfeeding?

- Yes (1)
- No (2)

Q12 Do you have a partner?

- Yes (1)
- No (2)

Q13 If you answered "yes" to question 12, does your partner support you with breastfeeding?

- Yes (1)
- No (2)

Q14 Do other people in your life (friends, family, etc.) support you with breastfeeding?

- Yes (1)
- No (2)

Q15 After your most recent birth experience, how long did you breastfeed?

________________________________________________________________

Q16 Are you still breastfeeding?

- Yes (1)
- No (2)

Q17 At any point after your most recent birth experience, did you breastfeed exclusively? (No supplementation with formula)

- Yes (1)
- No (2)

Q18 If you answered "yes" to the previous question, how long did you exclusively breastfeed? (If you are still exclusively breastfeeding, enter the number of months and write "ongoing.")

________________________________________________________________
